# A Case-Control Study Examining Disparities in Clinical Trial Participation Among Breast Surgical Oncology Patients

**DOI:** 10.1093/jncics/pkz103

**Published:** 2019-12-16

**Authors:** Oluwadamilola M Fayanju, Yi Ren, Samantha M Thomas, Rachel A Greenup, Terry Hyslop, E Shelley Hwang, John H Stewart

**Affiliations:** 1 Department of Surgery, Duke University School of Medicine, Durham, NC, USA; 2 Women’s Cancer Program, Duke Cancer Institute, Durham, NC, USA; 3 Department of Population Health Sciences, Duke University School of Medicine, Durham, NC, USA; 4 Duke Forge, Duke University, Durham, NC, USA; 5 Department of Surgery, Durham VA Medical Center, Durham, NC, USA; 6 Biostatistics Shared Resource, Duke Cancer Institute, Durham, NC, USA; 7 Department of Biostatistics and Bioinformatics, Duke University School of Medicine, Durham, NC, USA; 8 Department of Surgery, University of Illinois at Chicago, Chicago, IL, USA; 9 Department of Surgery, University of Illinois Cancer Center, Chicago, IL, USA

## Abstract

**Background:**

Clinical trial participation among racial and ethnic minorities remains low despite national efforts. We sought to determine how participation in clinical trials by breast surgical oncology patients has changed over time and what characteristics are associated with participation.

**Methods:**

Women with breast cancer enrolled in National Cancer Institute–sponsored, cooperative-group trials from 2000 to 2012 and who underwent oncologic surgery (n = 17 125) were compared with trial-eligible women in the National Cancer Database diagnosed in 2000–2012 (n = 792 719). Race-specific trial participation was plotted over time by income and reported as a proportion of the combined cohorts. Factors associated with trial participation were estimated using logistic regression; we report odds ratios (ORs) with 95% confidence intervals (CIs). A *P* value less than  .05 was considered statistically significant for all analyses. All tests were two-sided.

**Results:**

Participation declined across all groups over time because of a decrease in the scale and number of trials. In 2000–2003, Asian–Pacific Islander (7.17%), Hispanic (3.48%), and white (7.13%) patients from the highest income group had higher participation than their lower-income counterparts (Asian–Pacific Islander: 3.95%; Hispanic: 2.67%; white: 5.96%), but by 2008–2012, only high-income white patients participated more than lower-income whites (0.32% vs 0.25%, all *P* < .01). Black (OR = 0.80, 95% CI = 0.75 to 0.85) and Hispanic (OR = 0.84, 95% CI = 0.77 to 0.92) patients were less likely to participate than whites, but there were statistically significant interactions between income and race and ethnicity, with high-income black patients being approximately 50% less likely to participate than lower-income blacks (all *P* < .001).

**Conclusions:**

Multifaceted interventions addressing the intersectionality of race, ethnicity, and other patient characteristics are needed to address persistent disparities in trial participation among breast surgical oncology patients.

In 1993, the United States (US) Congress enacted the National Institutes of Health (NIH) Revitalization Act, which was conceived to encourage the participation of women as well as racial and ethnic minority patients in NIH-sponsored research (1). Universal access to clinical studies has since become a top priority of the National Cancer Institute (NCI), but research comparing trial enrollment among minorities with their representation in state and national cancer registries has yielded conflicting results (2–5).

Duma et al.’s (6) 2018 article reviewing 14 years of clinical trial enrollment in cancer patients confirmed that trial participation among racial and ethnic minorities remained disproportionately low across most disease sites. But the authors reported near equal participation by race and ethnicity among breast cancer trials and offered these trials as an example of how racial parity in trial participation might ideally be achieved. Notably, however, this review specifically excluded trials for which a surgical intervention was being tested.

Although there are breast cancer trials that examine the benefit of systemic therapy among patients who have also undergone surgery, it remains unclear whether the relative parity of participation seen among the breast cancer trials reviewed by Duma and colleagues would also be observed among trials in which oncologic surgery was the intervention being assessed or an important condition of enrollment. Furthermore, it is unknown to what extent socioeconomic factors might mediate racial and ethnic disparities in trial enrollment among breast surgical oncology patients given evidence that certain patient- and system-level factors—including access to postoperative rehabilitation and quality at the hospital level because of regionalization, respectively—might more specifically affect the treatment trajectory of patients who undergo oncologic surgery as compared with patients receiving medical therapy (7).

Accordingly, we sought to compare a contemporary cohort of breast surgical oncology patients—that is, patients for whom oncologic surgery is the intervention being tested or for which surgery is a criterion for participation—who were enrolled in clinical trials with a national sample of similar patients to assess patterns of trial participation over time, identify differences between trial participants and patients captured in institutional tumor registries, and determine which patient characteristics are associated with likelihood of trial participation.

## Methods

### Cohort

We sought to examine clinical trial participation among patients with breast cancer who underwent oncologic surgery. In December 2014, ClinicalTrials.gov was searched to identify trials conducted between 1999 and 2012 in the United States for patients 18 years and older diagnosed with breast cancer. Filters were used to identify trials that were conducted by oncology cooperative groups (American College of Surgeons Oncology Group, Cancer and Leukemia Group B, Eastern Cooperative Oncology Group [ECOG], North Central Cancer Treatment Group, National Surgical Adjuvant Breast and Bowel Project, and Southwest Oncology Group); were sponsored by the NCI; were phase II or III; included surgery as an intervention or treatment; and were completed or terminated as of December 2014, resulting in a total of 47 clinical trials. Participant data for these trials were requested from NCI’s Cancer Therapy Evaluation Program (CTEP), which maintains patient-level information about individuals enrolled in NCI-sponsored, cooperative-group trials. Data for patients enrolled from 2000 to 2012 in 14 of the requested trials were provided by CTEP in June 2016.

Individual trial participant data—including age at diagnosis, year of trial enrollment, race, and ethnicity—were provided in a deidentified format except for zip code, which was used to link individual participants to zip-code level, area-based socioeconomic indicators including annual median household income, proportion of residents with at least a high school (HS) education, and geographic location. Trial inclusion criteria and final enrollment figures were cross-verified using both ClinicalTrials.gov and the respective journal publications in which trial results were ultimately published. All but two of the included trials excluded men, and of these, one enrolled one man (8) (who was excluded from this study) and the other enrolled none (9).

Patients from the trial database enrolled in 2000–2012 were compared with women with breast cancer selected from the 1998–2012 National Cancer Database (NCDB) Participant User File who were diagnosed in 2000–2012 and were eligible for at least one trial. Because trial participation in the United States is approximately 3% (10), it was assumed for the purpose of this study that patients in the NCDB did not participate in any trials. For both cohorts, race and ethnicity were combined into one variable with six categories: Asian/Pacific Islander (API), non-Hispanic black, Hispanic, Native American, non-Hispanic white, and other.

### Statistical Analysis

Patient characteristics were summarized with N (%) for categorical variables and median (interquartile range) for continuous variables. χ^2^ and *t* tests were used to compare categorical and continuous variables, as appropriate. Unadjusted trial participation rates within each of the four largest racial and ethnic groups (API, non-Hispanic black, Hispanic, and non-Hispanic white) were plotted over time; the Breslow-Day procedure was used to test for homogeneity between income and trial participation while controlling for race, with a significant *P* value of less than .05 indicating differences across racial groups (11). All tests were two-sided. Multivariate logistic regression was performed for the binary outcome of trial participation (yes/no), while adjusting for age, race/ethnicity (a composite variable), number of trial enrollment slots per year (divided into three levels: <500, 500–1000, and >1000; this variable was included in place of year [of enrollment and diagnosis for CTEP trial and NCDB patients, respectively] to better capture how opportunity for enrollment changed over time), area-based education, area-based median household income, and geographic location. Two-way and three-way interaction terms for race/ethnicity, income, education, and enrollment slots per year were estimated. Because payor information was not available for clinical trial participants, a subgroup regression analysis was performed on patients aged 65 years and older to assess whether associations with trial participation in the full cohort would persist in a cohort of uniformly Medicare-eligible patients. Finally, a mediation analysis (12,13) was performed for the full cohort to determine to what, if any extent, interracial differences in trial participation were mediated by socioeconomic factors. We report odds ratios (ORs) and 95% confidence intervals (CIs) with a significance level of .05 for all analyses. Pairwise comparisons of odds ratios were conducted for significant interactions, and the Benjamini-Hochberg procedure was used to adjust for multiplicity (14). Only patients with available data were included in the regression models, and effective sample sizes are included in all tables. All statistical analyses were conducted using SAS 9.4 (SAS Institute, Cary, NC) and R 3.5.0 (R Foundation for Statistical Computing, Vienna, Austria). Our study was approved by the Duke University School of Medicine Institutional Review Board.

## Results

### Patient Characteristics

Our trial cohort included 17 124 patients with breast cancer, and 792 719 patients were included in the NCDB cohort (Table 1) based on eligibility for at least one of the 14 included trials, seven of which specifically examined surgical interventions (Table 2;Supplementary Table 1, available online). Within the NCDB control group, 469 111 patients (59.2%) were eligible for only one trial, 204 294 patients (25.8%) were eligible for two trials, and 119 314 patients (15.1%) were eligible for three trials. A higher proportion of trial participants were aged 65 years and older (38.1% vs 27.9%), white (83.5% vs 73.7%), and from areas with higher levels of educational attainment (eg, >93% HS education: 32.5% vs 27.4%) than the NCDB controls (both *P* < .001). Trial participants were also slightly younger (median = 58 vs 60 years, *P* < .001) and more likely to be from the Midwest (28.9% vs 25.4%). Among trial participants, we found that the median age of patients varied based on the ECOG eligibility criteria for a given trial: the median age in trials for which ECOG scores were required to be no more than 1 (an indicator of high functional status) was 51 years, whereas the median age of patients enrolled in trials with an ECOG cutoff of no more than 2 was 59 years and for those with no cutoff was 57 years.

**Table 1.** Characteristics of breast cancer patients in National Cancer Institute-sponsored surgical oncology trials and trial-eligible controls from the NCDB, 2000–2012*

Characteristics	All patients (%)(n = 809 843; 100%)	TDB (%)(n = 17 124; 2.1%)	NCDB (%)(n = 792 719; 97.9%)	*P*
Age, median (IQR)	60 (50–70)	58 (50–66)	60 (50–70)	<.001
Age group, y				
<40	45 125 (5.6)	44 273 (5.6)	852 (5)	<.001
40–64	457 895 (56.5)	446 406 (56.3)	11 489 (67.1)	
≥65	306 811 (37.9)	302 040 (38.1)	4771 (27.9)	
Race				
Non-Hispanic white	598 316 (73.9)	14 295 (83.5)	584 021 (73.7)	<.001
Non-Hispanic black	86 142 (10.6)	1254 (7.3)	84 888 (10.7)	
API	23 832 (2.9)	407 (2.4)	23 425 (3)	
Native American	2044 (0.3)	34 (0.2)	2010 (0.3)	
Hispanic	40 395 (5)	689 (4)	39 706 (5)	
Other	51 392 (6.3)	445 (2.6)	50 947 (6.4)	
MH annual income				
<$38 000	124 384 (15.4)	2210 (12.9)	122 174 (15.4)	<.001
$38 000–47 999	170 293 (21)	3387 (19.8)	166 906 (21.1)	
$48 000–62 999	211 569 (26.1)	4241 (24.8)	207 328 (26.2)	
≥$63 000	287 846 (35.5)	5648 (33)	282 198 (35.6)	
HS graduation				
≤79%	120 386 (14.9)	1722 (10.1)	118 664 (15)	<.001
79.1–87%	189 540 (23.4)	3060 (17.9)	186 480 (23.5)	
87.1–93%	262 112 (32.4)	5180 (30.2)	256 932 (32.4)	
>93%	222 457 (27.5)	5561 (32.5)	216 896 (27.4)	
Facility location				
West	141 946 (17.5)	2728 (15.9)	139 218 (17.6)	
Midwest	206 308 (25.5)	4948 (28.9)	201 360 (25.4)	<.001
Northeast	174 716 (21.6)	2397 (14)	172 319 (21.7)	
South	215 157 (26.6)	3690 (21.5)	211 467 (26.7)	
Unknown	71 716 (8.9)	3361 (19.6)	68 355 (8.6)	
Year				
2000–2003	176 815 (21.8)	11 909 (69.5)	164 906 (20.8)	<.001
2004–2007	122 845 (15.2)	3560 (20.8)	119 285 (15)	
2008–2012	510 183 (63)	1655 (9.7)	508 528 (64.1)	
Trial slots open at time of diagnosis/enrollment				
<500	411 188 (50.8)	1286 (7.5)	409 902 (51.7)	<.001
500–1000	179 111 (22.1)	2443 (14.3)	176 668 (22.3)	
>1000	219 544 (27.1)	13 395 (78.2)	206 149 (26)	

* *t* tests and χ^2^ tests were used to compare continuous and categorical characteristics, respectively, between groups. API = Asian/Pacific Islander; HS = high school; IQR= interquartile range; MH = median household; NCDB = National Cancer Database; TDB = trial database.

**Table 2.** National Cancer Institute–sponsored cooperative-group trials with breast surgical oncology patients enrolled between 2000 and 2012*

Trial	Phase	Surgical intervention	Year (start-completion date/published)	No. enrolled (published)	No. enrolled(CTEP)	Age, y	cT	cN	cM	Receptor	Breast surgery	Functional status	Sex	Previous malignancy allowed
ACOSOG-Z0010 (15)	III	Yes	1999–2003	5539	5267	≥18	1–2	0	0	All	Lumpectomy	ECOG ≤2	F	Yes
ACOSOG-Z0011 (16	III	Yes	1999–2004	891	846	≥18	1–2	0	0	All	Lumpectomy	ECOG ≤2	F	No
ACOSOG-Z1031 (17, 18)	III	No	2006–2009 (Cohort A) 2009–2011 (Cohort B)	A: 377 B: 255	622	≥18	2–4c	All	0	ER+	All	ECOG ≤2	F	No
ACOSOG-Z1041 (19)	III	No	2007–2011	282	282	≥18	cT1N1-3, cT2-4N0-1		0	HER2+	All	ECOG ≤1	F	No
ACOSOG-Z1071 (20)	II	Yes	2009–2011	756	757	≥18	0–4c	1–2	0	All	All	ECOG ≤1	F	Yes
ACOSOG-Z1072 (21)	II	Yes	2009–2013	99	99	≥18	1	All	0	All	All	N/A	F	No
ACOSOG-Z11102 (22)	II	Yes	2012–2016	223	189	≥40	1–2	0–1	0	All	Lumpectomy	ECOG ≤2	F	No
CALGB-40903 (23)	II	No	2012–2016	108	105	≥18	is/1mi	0	0	ER+ &/or PR+	All	ECOG ≤1	F	Yes
ECOG-2108 (8)	III	Yes	2011–2015	383	392	≥18	All	All	1	All	All	N/A	Both	No
NCCTG-N0338 (9)	II	No	2005–2007	57	57	≥18	2–4	1–3	0	All	All	ECOG ≤1	Both	No
NSABP-B-32 (24)	III	Yes	1999–2004	5611	5474	≥18	1-3	0	0	All	All	N/A	F	No
NSABP-B-35 (25)	III	No	2003–2006	3104	3104	≥18	is	0	0	ER+ and/or PR+	Lumpectomy	ECOG ≤2	F	No
SWOG-S0012 (17)	III	No	2001–2005	399	399	≥18	T3N0, T3N1-2, T0-2N2, T4N0-3, T0-4N3		0	All	All	ECOG ≤2	F	No
SWOG-S9927 (26)	III	No	2000–2003	98	98	≥21	1–2	1	0	All	Mastectomy	ECOG ≤1	F	No

* ACOSOG = American College of Surgeons Oncology Group; CALGB = Cancer and Leukemia Group B; cT = clinical tumor classification; CTEP = Cancer Therapy Evaluation Program; cN = clinical nodal classification; cM = clinical metastasis classification; ECOG = Eastern Cooperative Oncology Group; N/A = not applicable or available; NCCTG = North Central Cancer Treatment Group; NSABP = National Surgical Adjuvant Breast and Bowel Project; SWOG = Southwest Oncology Group.

After adjusting for known covariates, logistic regression demonstrated that higher level of area-based education (>93% vs ≤79% HS education: OR = 2.55, 95% CI = 2.37 to 2.75) and being from the Midwest (OR = 1.33, 95% CI = 1.27 to 1.40) or the South (OR = 1.15, 95% CI = 1.09 to 1.21) were associated with greater likelihood of clinical trial participation relative to less education and being from the West (all *P* < .001; Table 3). Patients younger than 40 years (OR = 0.70, 95% CI = 0.65 to 0.76) and those 65 years and older (OR = 0.59, 95% CI = 0.57 to 0.61) were less likely to participate than patients between the ages of 40 and 64 years (*P* < .001), while Hispanic (OR = 0.84, 95% CI = 0.77 to 0.92) and non-Hispanic black (OR = 0.80, 95% CI = 0.75 to 0.85) patients were less likely to participate than non-Hispanic white patients (*P* < .001). Patients from the highest area-based income bracket (≥$63 000) were less likely to participate than those from the lowest income bracket (<$38 000: OR = 0.63, 95% CI = 0.59 to 0.68), and likelihood of enrollment declined with increasing income (*P* < .001; Table 3). Not surprisingly, having more opportunities to participate (>1000 slots/year: OR = 20.03, 95% CI =18.88 to 21.24; and 500–1000 slots/year: OR = 4.40, 95% CI = 4.11 to 4.72) was also associated with higher likelihood of participation (vs <500 slots/year; *P* < .001). Among Medicare-eligible patients (ie, 65 years and older), whites continued to constitute the largest proportion of both trial and NCDB patients, and as observed in the full cohort, a higher proportion of the trial participants were white as compared with the NCDB controls (87.1% vs 78.7%; *P* < .001; Supplementary Table 2, available online). Also in keeping with the full cohort, higher area-level education and lower area-level income were associated with greater likelihood of trial participation (Supplementary Table 3, available online).

**Table 3.** Multivariate logistic regression on likelihood of trial participation of breast surgical oncology trial participants vs NCDB controls, 2000–2012*

Characteristics	OR (95% CI)	*P*	Overall *P*
Age group, y			
40–64	1.00 (Referent)		<.001
<40	0.70 (0.65 to 0.76)	<.001	
≥65	0.59 (0.57 to 0.61)	<.001	
Race/ethnicity			
Non-Hispanic white	1.00 (Referent)		<.001
Non-Hispanic black	0.80 (0.75 to 0.85)	<.001	
Hispanic	0.84 (0.77 to 0.92)	<.001	
API	0.93 (0.83 to 1.03)	.16	
Native American	0.72 (0.50 to 1.04)	.08	
Other	0.26 (0.23 to 0.29)	<.001	
HS graduation			
≤79%	1.00 (Referent)		<.001
79.1–87%	1.21 (1.13 to 1.29)	<.001	
87.1–93%	1.71 (1.60 to 1.82)	<.001	
>93%	2.55 (2.37 to 2.75)	<.001	
MH income			
<$38 000	1.00 (Referent)		<.001
$38 000–47 999	0.92 (0.86 to 0.97)	.005	
$48 000–62 999	0.80 (0.75 to 0.85)	<.001	
≥$63 000	0.63 (0.59 to 0.68)	<.001	
Facility location			
West	1.00 (Referent)		<.001
Midwest	1.33 (1.27 to 1.40)	<.001	
Northeast	0.74 (0.70 to 0.78)	<.001	
South	1.15 (1.09 to 1.21)	<.001	
Unknown	1.44 (1.35 to 1.53)	<.001	
Slots/year			
<500	1.00 (Referent)		<.001
500–1000	4.40 (4.11 to 4.72)	<.001	
>1000	20.03 (18.88 to 21.24)	<.001	

* Trial participants = 15 483; NCDB eligible controls = 771 101; no interaction terms. API = Asian/Pacific Islander; CI = confidence interval; HS = high school; MH = median household; NCDB = National Cancer Database; OR = odds ratio.

**Figure 1.**
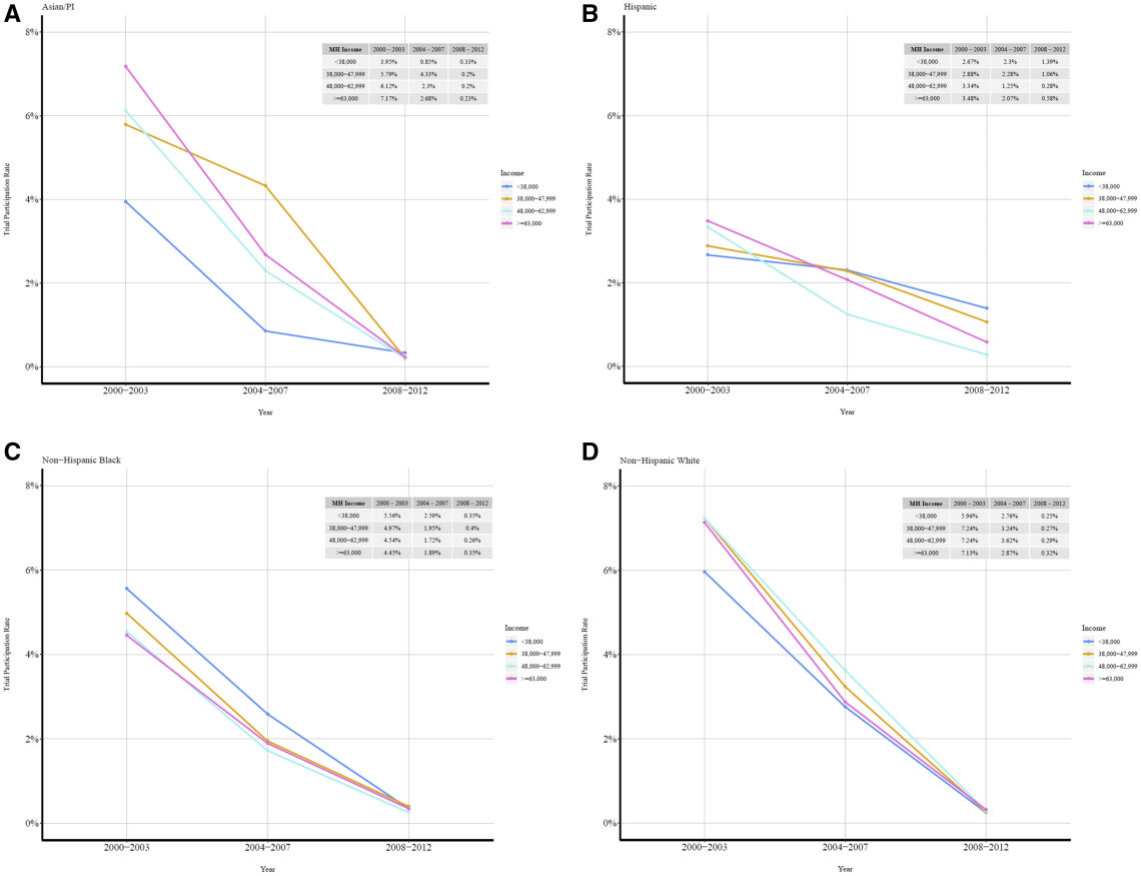
**A–D)** Unadjusted trial participation rates of breast cancer patients in National Cancer Institute–sponsored surgical oncology trials, 2000–2012. API = Asian/Pacific Islander; MH = median household.

To examine the association between lower area-based income and higher likelihood of trial participation, we plotted true, unadjusted trial participation rates by income group within each racial/ethnic group (Figure 1). A majority of trial participants were enrolled in the early 2000s, after which there was a decline in both the number and size of trials across all racial/ethnic groups and in relative participation rates across income groups (Figure 1; Table 1). In 2000–2003, when trial participation was the highest across all groups, API (7.17%), Hispanic (3.48%), and white (7.13%) patients from the highest income group (≥$63 000) had greater trial participation than their counterparts in the lowest income group (<$38 000; API = 3.95%, Hispanic = 2.67%, white = 5.96%; *P* = .003). However, by 2008–2012, participation had fallen drastically for all races and ethnicities (Figure 1); indeed, only white high-income patients had a higher unadjusted participation rate than their lower-income counterparts (0.32% vs 0.25%; *P* < .001), a finding that was statistically significant but of questionable clinical significance. Among black patients, trial participation was higher among low-income patients than high-income patients in 2000–2003 (5.56% vs 4.45%) and 2004–2007 (2.59% vs 1.89%), but these rates were equal by 2008–2012 (0.35% for both income groups; Figure 1).

In keeping with these changing patterns in participation rates, we found statistically significant interactions for race/ethnicity *education, income*education, slots per year*education, slots per year*race/ethnicity, income*race/ethnicity, slots per year*race and ethnicity, and income*race and ethnicity*slots per year of enrollment (CTEP trial cases)/diagnosis (NCDB controls) (*P* < .001; Tables 4 and 5). With decreased sample size for the Medicare-eligible population, estimation of interaction effects for this subgroup was not possible, so we only report the interactions for the full cohort.

**Table 4.** Multivariate logistic regression on likelihood of trial participation of breast surgical oncology trial participants vs NCDB controls, 2000–2012, with interaction terms

Characteristics	OR (95% CI)	*P*	Overall *P*
Age group, y			
40–64	1.00 (Referent)		<.001
<40	0.71 (0.65 to 0.76)	<.001	
≥65	0.59 (0.56 to 0.61)	<.001	
HS graduation			
≤79%	1.00 (Referent)		<.001
79.1–87%	1.23 (1.16 to 1.32)	<.001	
87.1–93%	1.74 (1.63 to 1.86)	<.001	
>93%	2.59 (2.40 to 2.79)	<.001	
MH income			
<$38 000	1.00 (Referent)		<.001
$38 000–47 999	0.77 (0.59 to 0.99)	.04	
$48 000–62 999	0.74 (0.58 to 0.95)	.02	
≥$63 000	0.64 (0.50 to 0.80)	<.001	
Facility location			
West	1.00 (Referent)		<.001
Midwest	1.33 (1.26 to 1.39)	<.001	
Northeast	0.73 (0.69 to 0.77)	<.001	
South	1.16 (1.10 to 1.22)	<.001	
Unknown	1.44 (1.35 to 1.54)	<.001	
Race/Ethnicity			
Non-Hispanic white	1.00 (Referent)		<.001
API	1.61 (0.51 to 5.09)	.42	
Hispanic	6.06 (4.45 to 8.25)	<.001	
Native American	2.95 (0.93 to 9.37)	.07	
Non-Hispanic black	1.30 (0.94 to 1.79)	.11	
Other	0.74 (0.36 to 1.52)	.41	
Slots per year			
<500	1.00 (Referent)		.99
500–1000	4.31 (3.34 to 5.55)	<.001	
>1000	21.62 (17.47 to 26.77)	<.001	
Income*slots per year			
	Interaction^†^		.01
Income*race/ethnicity			
	Interaction^†^		<.001
Slots per year *race/ethnicity			
	Interaction^†^		<.001
Income*race/ethnicity*slots per year			
	Interaction^†^		<.001

*Trial participants = 15 483; NCDB eligible controls = 771 101; three- and two-way-interactions included. API = Asian/Pacific Islander; CI = confidence interval; HS = high school; MH = median household; NCDB = National Cancer Database; OR = odds ratio.

† ORs for interactions are not shown. Select clinically relevant pairwise odds ratios are presented in Table 5.

**Table 5.** Odds ratio from pairwise comparisons across race and income, breast surgical oncology trial participants vs NCDB controls, 2000–2012*

Characteristics		OR (95% CI)	*P*	Adjusted *P*^†^
Racial difference at >1000 slots per year (given race vs non-Hispanic white)				
Race/ethnicity	MH income			
API	<$38 000	0.57 (0.29 to 1.12)	.10	.13
	≥$63 000	1.04 (0.88 to 1.23)	.62	.62
Hispanic	<$38 000	0.53 (0.41 to 0.69)	<.001	<.001
	≥$63 000	0.57 (0.44 to 0.73)	<.001	<.001
Non-Hispanic black	<$38 000	0.94 (0.84 to 1.06)	.30	.35
	≥$63 000	0.66 (0.54 to 0.79)	<.001	<.001
MH income difference (≥$63 000 vs <$38 000)				
Race/ethnicity	Slots/year			
API	<500	0.27 (0.08 to 0.95)	.04	.06
	>1000	1.24 (0.63 to 2.47)	.53	.57
Hispanic	<500	0.19 (0.12 to 0.29)	<.001	<.001
	>1000	0.73 (0.51 to 1.05)	.09	.13
Non-Hispanic black	<500	0.45 (0.28 to 0.74)	.002	.003
	>1000	0.48 (0.38 to 0.59)	<.001	<.001
Non-Hispanic white	<500	0.64 (0.51 to 0.81)	<.001	<.001
	>1000	0.68 (0.63 to 0.74)	<.001	<.001

* Derived from multivariate logistic regression model described in Table 4; hundreds of pairwise odds ratios were generated but only those of greatest clinical relevance are displayed. API = Asian/Pacific Islander; CI = confidence interval; MH = median household; NCDB = National Cancer Database; OR = odds ratio.

† Benjamini-Hochberg procedure was used to adjust *P* values for multiple comparisons.

Income-based interactions were incorporated into the final model, but the education-based interactions were not because their inclusion did not improve the overall fit of the model when the Akaike information criterion and Bayesian information criterion were considered together. As mentioned previously, likelihood of trial enrollment declined for all racial and ethnic groups over time, but within racial/ethnic and income groups, there were statistically significant differences.

After adjustment, when opportunity to participate was greatest (ie, >1000 slots/year), low-income Hispanic patients (OR = 0.53, CI = 0.41 to 0.69) were still approximately 50% less likely to participate than low-income whites, and high-income Hispanic (OR = 0.57, 95% CI = 0.44 to 0.73), and black (OR = 0.66, 95% CI = 0.54 to 0.79) patients were approximately 45% and 35% less likely, respectively, to participate than high-income whites (all *P* < .001; Table 5).

Among blacks, patients with high area-based income were approximately 55% less likely to participate than low-income patients regardless of how great (>1000 slots/year: OR = 0.48, 95% CI = 0.38 to 0.59) or small (<500 slots/year: OR = 0.45, 95% CI = 0.28 to 0.74) the opportunity to participate, whereas high-income whites were only approximately 30–35% less likely to participate than low-income whites, again, regardless of how much opportunity there was to participate (>1000 slots/year: OR = 0.68, 95% CI = 0.63 to 0.74; <500 slots/year: OR = 0.64, 95% CI = 0.51 to 0.81; *P* < .001 for all adjusted ORs; Table 5). When opportunity to participate was low (<500 slots/year), high-income Hispanic patients were more than 80% less likely to participate than their low-income counterparts (OR = 0.19, 95% CI = 0.12 to 0.29; *P* < .001; Table 5).

Our mediation analysis demonstrated that the effect of race was moderately reduced after adjusting for socioeconomic factors but remained statistically significant (see Supplementary Materials, available online), thus confirming that the effect of race on trial participation was partially mediated by socioeconomic factors.

## Discussion

In our examination of trial participation by breast surgical oncology patients, we found that during the 12-year inclusion period, black and Hispanic patients were less likely to participate in clinical trials than whites—who constituted more than 80% of trial participants overall—and that trial participation has declined across all racial and ethnic groups over time. However, our study yielded a mixed picture on the state of diversity with regard to trial participation among breast surgical oncology patients: area-based patient income was strongly associated with clinical trial participation but in varying ways and to different extents across racial and ethnic groups. Initially, high-income API, Hispanic, and white patients had higher rates of participation than their lower-income counterparts, but gains in participation appear to have been made among lower-income members of these groups, to the point that low-income API and Hispanic patients had higher rates of participation by the end of the study period relative to their higher-income counterparts. But among black patients, lower area-based income (as compared with higher income) was associated with higher rates of trial participation throughout the study period, although this difference disappeared in later years, when participation across all racial and ethnic groups had declined to extremely low levels. In this context of much lower participation overall, improved participation among low-income, non-black patients resulted in a statistically significant association between declining area-based income and increased likelihood of participation, thereby suggesting that low income was associated with higher trial participation throughout the 12-year period of study, when in fact this relationship was highly dynamic over time.

Our study is unique in demonstrating the complex interplay between race and income in a longitudinal assessment of disparities in clinical trial participation, and our results suggest the need for strategies to improve trial participation that account for the intersectionality of potential participants’ racial and socioeconomic characteristics. Based on these findings, one might conclude that efforts to diversify trial enrollment may have contributed to more low-income API, white, and Hispanic patients enrolling in later years than in years past. The increased likelihood over time of non-black, low-income, racial and ethnic minority patients’ participating in trials relative to their white counterparts may also reflect the fact that poverty among whites tends to be concentrated in rural areas that are also remote from urban sites of trial participation (27). In contrast, recruitment strategies targeting black patients will need to address the unique barriers faced not only by low-income blacks but also by their higher-income counterparts, who may have significant wariness of research participation as a result of having greater awareness of past wrongs and a greater desire and ability to exhibit choice in the type of care they receive (28). Finally, although Hispanic Americans represent the single largest growing demographic in the United States (29), their representation in clinical oncology trials remains low relative to their proportion of the population (3,4,6,29,30), a state of affairs that can be ascribed to previously identified barriers including failure to provide translated education material as well as exclusions related to insurance status (30).

If clinical trial participation is to reflect the makeup of an increasingly diverse society whose members are living longer and with more comorbidities, efforts must go beyond simply making trials more logistically accessible for patients. In our study, trials with more stringent requirements about patient functional status (as defined by ECOG score) had younger patients, raising concerns for systematic exclusion of older patients. Furthermore, exclusion criteria that include functional status and/or comorbidities that are disproportionately found in people of color could further contribute to their underrepresentation in clinical trials (30). In much the same way that it is difficult and even dangerous to apply medications that are solely tested and developed in men to the treatment of women, structural features of trial design that contribute disproportionately to low inclusion of racial and ethnic minorities compromise our collective ability to apply the results of oncology innovation to diverse populations.

Over the course of our study period, trial participation declined sharply, and this phenomenon reflects both a shift to smaller and fewer trials over time and to the fact that the number of patients contributed to the NCDB increased over the period of study. Thus, the numerator for our trial participation rate decreased (Figure 1) while our control group and, accordingly, our denominator increased (Table 1), making the overall rates lower and lower over time. Nevertheless, we have little reason to believe that this increase in the size of the NCDB had a clinically significant impact on our overall results because when we examined the racial and ethnic composition of the database, it remained fairly constant, with a small trend toward greater inclusion of non-white patients (23% of potentially trial-eligible patients in 2000–2003 vs 26% in 2008–2012) over time.

Notably, the declining number of large trials we report reflects an evolution in our collective understanding of breast cancer, which is increasingly recognized to be a heterogeneous array of diseases that share an anatomic location rather than a single condition. Earlier trials in breast surgical oncology—such as the Z0010, Z0011, and National Surgical Adjuvant Breast and Bowel Project-B32 trials, which all opened in 1999—enrolled breast cancer patients with limited to no information on biomarker status and limited ability to tailor treatment according to the biology of the tumor and its susceptibility to available systemic treatment. Today, we know that extent of surgery (eg, axillary lymph node sampling) can be tailored based not only on extent of disease but also on the anticipated efficacy of adjuvant therapy (eg, radiation). Indeed, there is ongoing work to determine whether exceptional responders to neoadjuvant systemic therapy with HER2-enriched and triple-negative cancers can avoid surgery altogether (31). With increasingly narrow, subtype-specific inclusion criteria, future trials designed to help us refine and de-escalate breast cancer treatment will necessarily be smaller than the trials of the past. Thus, we must strive not so much to have ever more opportunities for trial participation but rather to make sure that the right patients find their way to the right trials. Specifically, we must prioritize the recruitment and inclusion of patients of color, who are disproportionately affected by some of the most aggressive breast cancer subtypes, lest we prevent these patients from being a part of the scientific process to which they would make important contributions and from which they can directly benefit.

Our study had several limitations, including selection bias and an inability to account for patient preference, that are associated with conducting retrospective analyses of pooled cancer registries such as the NCDB. We also acknowledge limitations related to the types of information included in the datasets that were available to us. We realize these limitations are only partially accounted for through statistical methodology, and we describe and address them in detail as part of the Supplementary Materials (available online) accompanying this article. Finally, our study included only patients in NCI-sponsored clinical trials, the enrollment levels for which have declined over time while enrollment in non–NCI-sponsored (typically, industry-sponsored) oncologic clinical trials has increased (32). But with this increase in industry-sponsored investigations, we feel that the implications of our findings are especially important, because they can be applied to address concerns that industry-sponsored trials disproportionately target and enroll vulnerable groups who have limited or no other sources of health care (33).

In summary, our study demonstrated that racial and ethnic disparities persist with regards to trial participation among breast surgical oncology patients, with black and Hispanic patients being less likely to participate in trials than whites, but these differences are mediated by socioeconomic factors. We also found that the likelihood of trial participation has declined across all racial and ethnic groups over time but that gains in participation among low-income API, Hispanic, and white patients appear to have occurred. Black patients were the only group for whom lower income was consistently associated with higher rates of participation, but over time, participation rates have converged for all groups such that intraracial, socioeconomic differences in participation have become smaller but also multidirectional. As a result, trial participation for the 12-year period of our study was associated with white race; higher levels of area-based education; and, most notably, lower levels of area-based income, but these associations bely the complex and dynamic relationship of intersecting demographic characteristics over time. Thus, although racial and ethnic disparities in trial participation persist among breast surgical oncology patients, interventions to ensure equitable trial access and participation will need to be similarly diverse and multifaceted.

## Funding

This work is supported by the National Institutes of Health (Grant Number 1K08CA241390 [PI: Fayanju] to OMF), the National Center for Advancing Translational Sciences of the National Institutes of Health (Grant Number 1KL2TR002554 [PI: Svetkey] to OMF); the National Institutes of Health Building Interdisciplinary Research Careers in Women’s Health (Grant Number K12HD043446 [PI: Andrews] to RAG); the National Institutes of Health Cancer Clinical Investigator Team Leadership Award at the Wake Forest University School of Medicine (Grant Number P30CA012197 [PI: Pasche] to JHS); and the National Cancer Institute at the National Institutes of Health (P30CA014236 [PI: Kastan] to Duke Cancer Institute).

## Notes

The authors have no any conflicts of interest, financial or otherwise, to disclose. The NCDB is a joint project of the Commission on Cancer (CoC) of the American College of Surgeons and the American Cancer Society. The CoC’s NCDB and the hospitals participating in the CoC NCDB are the source of the de-identified data used herein; they have not verified and are not responsible for the statistical validity of the data analysis or the conclusions derived by the authors.

The content is solely the responsibility of the authors and does not necessarily represent the official views of the NIH.

The funders had no role in the design of the study; the collection, analysis, and interpretation of the data; the writing of the manuscript; and the decision to submit the manuscript for publication.

## Supplementary Material

pkz103_Supplementary_Data
