# Correction to: A Case-Control Study Examining Disparities in Clinical Trial Participation Among Breast Surgical Oncology Patients

**DOI:** 10.1093/jncics/pkaf076

**Published:** 2025-07-29

This is a correction to: Oluwadamilola M. Fayanju, Yi Ren, Samantha M. Thomas, Rachel A. Greenup, Terry Hyslop, E. Shelley Hwang, John H. Stewart IV, A Case-Control Study Examining Disparities in Clinical Trial Participation Among Breast Surgical Oncology Patients, *JNCI Cancer Spectrum*, Volume 4, Issue 2, April 2020, pkz103, https://doi.org/10.1093/jncics/pkz103.

In October 2024, a reader contacted the journal about a possible error in Table 1. After looking at their raw data, the authors confirmed that the data for Age Group (<40, 40-64, and ≥65 y) in the trial database (TDB) and National Cancer Database (NCDB) columns were inadvertently transposed. This error occurred when the authors were preparing a revised table.

**Table 1.** Characteristics of breast cancer patients in National Cancer Institute-sponsored surgical oncology trials and trial-eligible controls from the NCDB, 2000-2012*

Characteristics	All Patients (%) (n = 809 843; 100%)	TDB (%) (n = 17 124; 2.1%)	NCDB (%) (n = 792 719; 97.9%)	*P*
Age, median (IQR)	60 (50 - 70)	58 (50 - 66)	60 (50 - 70)	<.001
Age group, y				
<40	45 125 (5.6)	852 (5)	44 273 (5.6)	<.001
40-64	457 895 (56.5)	11 489 (67.1)	446 406 (56.3)	
≥65	306 811 (37.9)	4 771 (27.9)	302 040 (38.1)	
Race				
Non-Hispanic white	598 316 (73.9)	14 295 (83.5)	584 021 (73.7)	<.001
Non-Hispanic black	86 142 (10.6)	1 254 (7.3)	84 888 (10.7)	
API	23 832 (2.9)	407 (2.4)	23 425 (3)	
Native American	2 044 (0.3)	34 (0.2)	2 010 (0.3)	
Hispanic	40 395 (5)	689 (4)	39 706 (5)	
Other	51 392 (6.3)	445 (2.6)	50 947 (6.4)	
MH annual income				
<$38 000	124 384 (15.4)	2 210 (12.9)	122 174 (15.4)	<.001
$38 000-47 999	170 293 (21)	3 387 (19.8)	166 906 (21.1)	
$48 000-62 999	211 569 (26.1)	4 241 (24.8)	207 328 (26.2)	
≥$63 000	287 846 (35.5)	5 648 (33)	282 198 (35.6)	
HS graduation				
≤79%	120 386 (14.9)	1 722 (10.1)	118 664 (15)	<.001
79.1-87%	189 540 (23.4)	3 060 (17.9)	186 480 (23.5)	
87.1-93%	262 112 (32.4)	5 180 (30.2)	256 932 (32.4)	
>93%	222 457 (27.5)	5 561 (32.5)	216 896 (27.4)	
Facility location				
West	141 946 (17.5)	2 728 (15.9)	139 218 (17.6)	
Midwest	206 308 (25.5)	4 948 (28.9)	201 360 (25.4)	<.001
Northeast	174 716 (21.6)	2 397 (14)	172 319 (21.7)	
South	215 157 (26.6)	3 690 (21.5)	211 467 (26.7)	
Unknown	71 716 (8.9)	3 361 (19.6)	68 355 (8.6)	
Year				
2000-2003	176 815 (21.8)	11 909 (69.5)	164 906 (20.8)	<.001
2004-2007	122 845 (15.2)	3 560 (20.8)	119 285 (15)	
2008-2012	510 183 (63)	1 655 (9.7)	508 528 (64.1)	
Trial slots open at time of diagnosis/enrollment				
<500	411 188 (50.8)	1 286 (7.5)	409 902 (51.7)	<.001
500-1000	179 111 (22.1)	2 443 (14.3)	176 668 (22.3)	
>1000	219 544 (27.1)	13 395 (78.2)	206 149 (26)	

* t-tests and χ^2^ tests were used to compare continuous and categorical characteristics, respectively, between groups. API = Asian/Pacific Islander; HS=high school; MH=median household; IQR=interquartile range; TDB=trial database.

A corrected Table 1 is included in this notice in which the same data are presented in the correct columns.

A correction to the Results section is also necessary. The sentences, “A higher proportion of trial participants were aged 65 years and older (38.1% vs 27.9%), white (83.5% vs 73.7%), and from areas with higher levels of educational attainment (eg, >93% HS education: 32.5% vs 27.4%) than the NCDB controls (both *P* < .001). Trial participants were also slightly younger (median = 58 vs 60 years, *P* < .001) and more likely to be from the Midwest (28.9% vs 25.4%)” should be corrected to “A higher proportion of trial participants were white (83.5% vs 73.7%) and from areas with higher levels of educational attainment (eg, >93% HS education: 32.5% vs 27.4%) than the NCDB controls (both *P* < .001). Trial participants were also slightly younger (median = 58 vs 60 years), with a lower proportion of patients aged 65 years and older (27.9% vs 38.1%), and they were more likely to be from the Midwest (28.9% vs 25.4%, all *P* < .001).”

The authors regret the error.

These details have been corrected only in this correction notice to preserve the published version of record.

