# Supplementary material for: A Case-Control Study Examining Disparities in Clinical Trial Participation Among Breast Surgical Oncology Patients
Source: JNCI Cancer Spectr. 2019 Dec 16;4(2):pkz103. doi: 10.1093/jncics/pkz103 (PMC7083236; doi:10.1093/jncics/pkz103)
Supplement: pkz103_Supplementary_Data [file pkz103_supplementary_data.docx]

Fayanju OM, et al. A Case-control Study Examining Disparities in Clinical Trial Participation among Breast Surgical Oncology Patients

Online Supplement

**Supplemental Table 1. NCI-sponsored Cooperative-Group Trials with Breast Surgical Oncology Patients Enrolled between 2000-2012 with Itemized Study Interventions***

| Trial | Study Title  (from ClinicalTrials.gov) | Phase | Surgical Intervention? | Study Intervention | Year (Start-Completion Date/ Published) |
| --- | --- | --- | --- | --- | --- |
| ACOSOG-Z0010^1^ | A Prognostic Study of Sentinel Node and Bone Marrow Micrometastases in Women With Clinical T1-2 N0 Breast Cancer | III | Yes | 1 arm: Breast-conserving therapy (lumpectomy + radiation), sentinel lymph node biopsy, bone marrow aspiration from iliac crests | 1999-2003 |
| ACOSOG-Z0011^2^ | A Randomized Trial of Axillary Node Dissection in Women With Clinical T1-2 N0-1 M0 Breast Cancer Who Have a Positive Sentinel Node | III | Yes | 2 arms: Breast-conserving surgery (lumpectomy + radiation), sentinel lymph node biopsy vs axillary lymph node dissection | 1999-2004 |
| ACOSOG-Z1031^3,4^ | A Randomized Phase III Trial Comparing 16 to 18 Weeks of Neoadjuvant Exemestane (25 mg Daily), Letrozole (2.5 mg), or Anastrozole (1 mg) in Postmenopausal Women With Clinical Stage II and III Estrogen Receptor Positive Breast Cancer | III | No | 3 arms: Exemestane vs letrozole vs anastrozoleafter breast surgery with or without axillary lymph node dissection | 2006-2009 (Cohort A) 2009-2011 (Cohort B) |
| ACOSOG-Z1041^5^ | A Randomized Phase III Trial Comparing a Neoadjuvant Regimen of FEC-75 Followed by Paclitaxel Plus Trastuzumab With a Neoadjuvant Regimen of Paclitaxel Plus Trastuzumab Followed by FEC-75 Plus Trastuzumab in Patients With HER-2 Positive Operable Breast Cancer | III | No | 2 arms: FEC-75 then Paclitaxel/trastuzumab  vs Paclitaxel/trastuzumab then trastuzumab/FEC-75 prior to surgical resection | 2007-2011 |
| ACOSOG-Z1071^6^ | A Phase II Study Evaluating the Role of Sentinel Lymph Node Surgery and Axillary Lymph Node Dissection Following Preoperative Chemotherapy in Women With Node Positive Breast Cancer (T1-4, N1-2, M0) at Initial Diagnosis | II | Yes | 1 arm: Mastectomy or lumpectomy, both sentinel lymph node surgery and axillary lymph node dissection within 12 weeks of completing neoadjuvant chemotherapy | 2009-2011 |
| ACOSOG-Z1072^7^ | A Phase II Trial Exploring the Success of Cryoablation Therapy in the Treatment of Invasive Breast Carcinoma | II | Yes | 1 arm: Surgical resection (lumpectomy or mastectomy) and sentinel lymph node biopsy and/or axillary dissection within 28 days after completion of cryoablation | 2009-2013 |
| ACOSOG-Z11102^8^ | Impact of Breast Conservation Surgery on Surgical Outcomes and Cosmesis in Patients With Multiple Ipsilateral Breast Cancers (MIBC) | II | Yes | 1 arm: Breast-conserving therapy (lumpectomy + radiation), +/- chemotherapy and endocrine therapy as indicated | 2012-2016 |
| CALGB-40903^9^ | Phase II Study of Neoadjuvant Letrozole for Postmenopausal Women With Estrogen Receptor Positive Ductal Carcinoma In Situ (DCIS) | II | No | 1 arm: Letrozole followed by lumpectomy or mastectomy | 2012-2016 |
| ECOG-2108^10^ | A Randomized Phase III Trial of the Value of Early Local Therapy for the Intact Primary Tumor in Patients With Metastatic Breast Cancer | III | Yes | 2 arms: Upfront breast-conserving therapy (BCT) or total mastectomy vs symptom-based standard palliative therapy (to include radiotherapy alone, surgery alone, or a combination of both) | 2011-2015 |
| NCCTG-N0338^11^ | Phase II Trial of Docetaxel and Carboplatin Administered Every Two Weeks as Induction Therapy for Stage II or III Breast Cancer | II | No | 1 arm: Neoadjuvant docetaxel, carboplatin, and pegfilgrastim followed by definitive surgery | 2005-2007 |
| NSABP-B-32^12^ | A Randomized, Phase III Clinical Trial to Compare Sentinel Node Resection to Conventional Axillary Dissection in Clinically Node-Negative Breast Cancer Patients | III | Yes | 2 arms: Axillary lymph node dissection vs sentinel lymph node biopsy (with axillary lymph node dissection only if sentinel lymph node biopsy) | 1999-2004 |
| NSABP-B-35^13^ | A Clinical Trial Comparing Anastrozole With Tamoxifen in Postmenopausal Patients With Ductal Carcinoma in Situ (DCIS) Undergoing Lumpectomy With Radiation Therapy | III | No | 2 arms: Anastrozole vs tamoxifen following breast-conserving therapy (lumpectomy + radiation) | 2003-2006 |
| SWOG-S0012^3^ | A Comparative Randomized Study of Standard Doxorubicin and Cyclophosphamide Followed by Weekly Paclitaxel Vs. Weekly Doxorubicin and Daily Oral Cyclophosphamide Plus G-CSF Followed by Weekly Paclitaxel As Neoadjuvant Therapy For Inflammatory and Locally Advanced Breast Cancer | III | No | 2 arms: Neoadjuvant doxorubicin and cyclophosphamide followed by paclitaxel vs neoadjuvant weekly doxorubicin and daily cyclophosphamide with filgrastim followed by paclitaxel | 2001-2005 |
| SWOG-S9927^14^ | Randomized Trial of Post-Mastectomy Radiotherapy in Stage II Breast Cancer in Women With One to Three Positive Axillary Nodes Phase III | III | No | 2 arms: Adjuvant post-mastectomy radiation vs  no post-mastectomy radiation | 2000-2003 |

*ACOSOG, American College of Surgeons Oncology Group. CALGB, Cancer and Leukemia Group B. ECOG, Eastern Cooperative Oncology Group. NCCTG, North Central Cancer Treatment Group. NSABP, National Surgical Adjuvant Breast and Bowel Project. SWOG, Southwest Oncology Group.

Analyses for Patients ≥65 (i.e., Medicare-eligible)

**Supplemental Table 2. Characteristics of Breast Cancer Patients ≥65 years of age in NCI-sponsored Surgical Oncology Trials and Trial-eligible Controls from the National Cancer Data Base (NCDB), 2000-2012**

|  | | **All Patients** | **TDB** | **NCDB** |  |
| --- | --- | --- | --- | --- | --- |
|  | | **N=306,811**  **(100%)** | **N=4,771**  **(1.6%)** | **N=302,040**  **(98.4%)** | **P-Value** |
| Age | Median (IQR) | 74 (69 - 80) | 70 (67 - 75) | 74 (69 - 80) | <0.001 |
| Race | Non-Hispanic White | 241,981 (78.9%) | 4,157 (87.1%) | 237,824 (78.7%) | <0.001 |
|  | Non-Hispanic Black | 25,383 (8.3%) | 294 (6.2%) | 25,089 (8.3%) |  |
|  | Asian/PI | 5,751 (1.9%) | 69 (1.4%) | 5,682 (1.9%) |  |
|  | Native American | 502 (0.2%) | 2 (0%) | 500 (0.2%) |  |
|  | Hispanic | 10,237 (3.3%) | 136 (2.9%) | 10,101 (3.3%) |  |
|  | Other | 20,309 (6.6%) | 113 (2.4%) | 20,196 (6.7%) |  |
| MH Income | <$38,000 | 49,301 (16.1%) | 706 (14.8%) | 48,595 (16.1%) | 0.01 |
|  | $38,000-47,999 | 69,099 (22.5%) | 1,074 (22.5%) | 68,025 (22.5%) |  |
|  | $48,000-62,999 | 82,234 (26.8%) | 1,269 (26.6%) | 80,965 (26.8%) |  |
|  | ≥$63,000 | 100,515 (32.8%) | 1,396 (29.3%) | 99,119 (32.8%) |  |
| HS Graduation | ≤79% | 44,537 (14.5%) | 507 (10.6%) | 44,030 (14.6%) | <0.001 |
|  | 79.1-87% | 74,232 (24.2%) | 941 (19.7%) | 73,291 (24.3%) |  |
|  | 87.1-93% | 102,279 (33.3%) | 1,507 (31.6%) | 100,772 (33.4%) |  |
|  | >93% | 80,265 (26.2%) | 1,504 (31.5%) | 78,761 (26.1%) |  |
| Facility Location | West | 51,889 (16.9%) | 794 (16.6%) | 51,095 (16.9%) | <0.001 |
|  | Midwest | 80,597 (26.3%) | 1,350 (28.3%) | 79,247 (26.2%) |  |
|  | Northeast | 68,387 (22.3%) | 740 (15.5%) | 67,647 (22.4%) |  |
|  | South | 80,884 (26.4%) | 1,102 (23.1%) | 79,782 (26.4%) |  |
|  | Unknown | 25,054 (8.2%) | 785 (16.5%) | 24,269 (8%) |  |
| Year | 2000-2003 | 72,055 (23.5%) | 3,265 (68.4%) | 68,790 (22.8%) | <0.001 |
|  | 2004-2007 | 40,371 (13.2%) | 1,133 (23.7%) | 39,238 (13%) |  |
|  | 2008-2012 | 194,385 (63.4%) | 373 (7.8%) | 194,012 (64.2%) |  |
| Slots Open at Dx/Enroll | <500 | 154,127 (50.2%) | 359 (7.5%) | 153,768 (50.9%) | <0.001 |
|  | 500-1000 | 65,054 (21.2%) | 712 (14.9%) | 64,342 (21.3%) |  |
|  | >1000 | 87,630 (28.6%) | 3,700 (77.6%) | 83,930 (27.8%) |  |

HS, high school. MH, median household. IQR, interquartile range. PI, Pacific Islander. TDB, trial database.

**Supplemental Table 3. Multivariate Logistic Regression on Likelihood of Trial Participation of Breast Surgical Oncology Trial Participants vs NCDB Controls, Patients ≥65 years of age, 2000-2012**

|  | **OR (95% CI)** | **P-Value** | **Overall P-Value** |
| --- | --- | --- | --- |
| Age | | | |
| Continuous | 0.92 (0.91 - 0.92) | <0.001 |  |
| Race/Ethnicity | | | |
| Non-Hispanic White | -REF- |  | <0.001 |
| Asian/PI | 0.86 (0.66 - 1.12) | 0.264 |  |
| Hispanic | 0.89 (0.73 - 1.08) | 0.252 |  |
| Native American | 0.24 (0.06 - 0.96) | 0.044 |  |
| Non-Hispanic Black | 0.85 (0.75 - 0.96) | 0.010 |  |
| Other | 0.25 (0.20 - 0.30) | <0.001 |  |
| Facility Location | | | |
| West | -REF- |  | <0.001 |
| Midwest | 1.21 (1.10 - 1.32) | <0.001 |  |
| Northeast | 0.78 (0.70 - 0.86) | <0.001 |  |
| South | 1.15 (1.05 - 1.27) | 0.004 |  |
| Unknown | 1.36 (1.21 - 1.53) | <0.001 |  |
| MH Income | | | |
| <$38,000 | -REF- |  | <0.001 |
| $38,000-47,999 | 0.891 (0.802 - 0.99) | 0.032 |  |
| $48,000-62,999 | 0.761 (0.681 - 0.85) | <0.001 |  |
| ≥$63,000 | 0.54 (0.477 - 0.612) | <0.001 |  |
| HS Graduation | | | |
| ≤79% | -REF- |  | <0.001 |
| 79.1-87% | 1.254 (1.116 - 1.408) | <0.001 |  |
| 87.1-93% | 1.759 (1.557 - 1.986) | <0.001 |  |
| >93% | 2.865 (2.504 - 3.278) | <0.001 |  |
| Slots/year | | | |
| <500 | -REF- |  | <0.001 |
| 500-1000 | 4.967 (4.364 - 5.653) | <0.001 |  |
| >1000 | 21.119 (18.892 - 23.61) | <0.001 |  |

**With decreasing sample size of the ≥65-year-old subgroup analysis, we could not produce Type III p-values for the interactions conducted for the full cohort. But the odds ratio trends for the ≥65 patients mirror those in the model for the full cohort.**

Mediation Analysis

**In Table 1, we demonstrated that there is an association between race and likelihood of participation, with a higher proportion of white patients (83.5%) in the Trial Database (TDB) cohort as compared to the NCDB control group (73.7%, p<0.001), and this association with race was also observed in the multivariate logistic regression model (Table 3).**

**To determine whether the effect of race on likelihood of trial participation was mediated by socioeconomic factors (education and income), we first confirmed that race is associated univariately with the socioeconomic factors of education and income via Chi-square tests:**

|  | | **All Patients** | **Asian PI** | **Hispanic** | **Native American** | **Non-Hispanic Black** | **Non-Hispanic White** | **Other** |  |
| --- | --- | --- | --- | --- | --- | --- | --- | --- | --- |
|  | | **N=802,121** | 23,832 (2.9%) | 40,395 (5%) | 2,044 (0.3%) | 2,044  (0.3%) | 598,316 (73.9%) | 51,392 (6.3%) | **P-Value** |
| Median Household Income | ≥$63,000 | 287,846 (35.5%) | 13,352 (56%) | 9,764 (24.2%) | 395 (19.3%) | 14,392 (16.7%) | 230,602 (38.5%) | 15,965 (31.1%) | <0.01 |
|  | $38,000-47,999 | 170,293 (21%) | 2,902 (12.2%) | 9,260 (22.9%) | 456 (22.3%) | 19,046 (22.1%) | 125,305 (20.9%) | 12,009 (23.4%) |  |
|  | $48,000-62,999 | 211,569 (26.1%) | 5,726 (24%) | 10,761 (26.6%) | 465 (22.7%) | 16,801 (19.5%) | 161,924 (27.1%) | 13,897 (27%) |  |
|  | <$38,000 | 124,384 (15.4%) | 1,366 (5.7%) | 9,878 (24.5%) | 695 (34%) | 34,419 (40%) | 68,565 (11.5%) | 8,642 (16.8%) |  |
| HS Graduation | >93% | 222,457 (27.5%) | 7,492 (31.4%) | 4,543 (11.2%) | 345 (16.9%) | 8,111 (9.4%) | 184,864 (30.9%) | 14,555 (28.3%) | <0.01 |
|  | 79.1-87% | 189,540 (23.4%) | 4,575 (19.2%) | 8,885 (22%) | 551 (27%) | 30,715 (35.7%) | 130,748 (21.9%) | 12,513 (24.3%) |  |
|  | 87.1-93% | 262,112 (32.4%) | 7,171 (30.1%) | 7,594 (18.8%) | 625 (30.6%) | 18,717 (21.7%) | 207,517 (34.7%) | 17,990 (35%) |  |
|  | ≤79% | 120,386 (14.9%) | 4,113 (17.3%) | 18,654 (46.2%) | 493 (24.1%) | 27,146 (31.5%) | 63,585 (10.6%) | 5,484 (10.7%) |  |

**We then re-ran the regression model for the outcome “likelihood of trial participation” but excluded the socioeconomic factors (education, income):**

|  | **OR (95% CI)** | **P-Value** | **Overall P-Value** |
| --- | --- | --- | --- |
| Age | | | |
| Continuous | 0.985 (0.984 - 0.987) | <0.001 |  |
| Race/Ethnicity | | | |
| Non-Hispanic White | -REF- |  | <0.001 |
| Asian/PI | 0.837 (0.752 - 0.933) | 0.001 |  |
| Hispanic | 0.670 (0.613 - 0.732) | <0.001 |  |
| Native American | 0.681 (0.472 - 0.982) | 0.039 |  |
| Non-Hispanic Black | 0.686 (0.645 - 0.728) | <0.001 |  |
| Other | 0.262 (0.236 - 0.291) | <0.001 |  |
| Facility Location | | | |
| West | -REF- |  | <0.001 |
| Midwest | 1.394 (1.327 - 1.464) | <0.001 |  |
| Northeast | 0.726 (0.686 - 0.769) | <0.001 |  |
| South | 1.086 (1.031 - 1.143) | 0.002 |  |
| Unknown | 1.311 (1.232 - 1.396) | <0.001 |  |
| Slots/Year | | | |
| <500 | -REF- |  | <0.001 |
| 500-1000 | 4.375 (4.084 - 4.687) | <0.001 |  |
| >1000 | 19.644 (18.525 - 20.83) | <0.001 |  |

**Comparing the regression model above to the more complete model in Table 3 (which includes the two socioeconomic factors of education and income) demonstrates that the effect of race is moderately reduced after adjusting for socoioeconomic factors but remains significant. Thus, a partial mediation was found. We further investigated this finding by performing a causal mediation analysis with bootstrapping to estimate the average causal mediation effect (ACME), which was found to be significant (p<0.001):**

Causal Mediation Analysis

Nonparametric Bootstrap Confidence Intervals with the Percentile Method

Estimate 95% CI Lower 95% CI Upper p-value

ACME (control) -0.001085 -0.001389 0.00 <2e-16 ***

ACME (treated) -0.000135 -0.000203 0.00 <2e-16 ***

ADE (control) -0.015865 -0.017008 -0.01 <2e-16 ***

ADE (treated) -0.014915 -0.016175 -0.01 <2e-16 ***

Total Effect -0.016000 -0.017090 -0.01 <2e-16 ***

Prop. Mediated (control) 0.067820 0.048421 0.09 <2e-16 ***

Prop. Mediated (treated) 0.008462 0.004352 0.01 <2e-16 ***

ACME (average) -0.000610 -0.000779 0.00 <2e-16 ***

ADE (average) -0.015390 -0.016597 -0.01 <2e-16 ***

Prop. Mediated (average) 0.038141 0.027121 0.05 <2e-16 ***

---

Signif. codes: 0 ‘***’ 0.001 ‘**’ 0.01 ‘*’ 0.05 ‘.’ 0.1 ‘ ’ 1

Sample Size Used: 786584

Simulations: 500

**Accordingly, our mediation analysis supports our statement that interracial differences in trial participation were mediated by socioeconomic factors (Baron RM, Kenny DA. The moderator-mediator variable distinction in social psychological research: conceptual, strategic, and statistical considerations. J Pers Soc Psychol. 1986;51(6):1173-1182; Tingley D, Yamamoto T, Hirose K, Keele L, Imai K. Mediation: R Package for Causal Mediation Analysis. 2014;59(5):38).**

Study Limitations

**Our study had several limitations, which we allude to in the manuscript and expand upon below.**

**First, we assumed that the NCDB does not contain trial participants, even as we realize that a very small proportion of NCDB patients will, in fact, have participated in a clinical trial. Because Commission on Cancer (CoC) accreditation requires fulfilment of stringent criteria and requirements – including the availability of oncology specialists, critical ancillary services, and multidisciplinary cancer conferences (Bilimoria KY, et al. JCO 2009; 27(25):4177-4181), characteristics that have been shown to be associated with increased likelihood of trial enrollment (Frayne SM, et al. J Natl Med Assoc. 2001;93(11):450–457; Mahmud A, et al. Curr Oncol. 2018 Apr;25(2):119-125; Kehl KL, J Oncol Pract. 2015 May;11(3):e267-78)– it can be inferred that receiving treatment at a CoC-designated center (as all patients in the NCDB cohort did) may increase the likelihood of enrollment in a clinical trial as compared with receiving care at a site that is not CoC-accredited. But we felt that our assumption regarding these controls was reasonable given low national rates of trial participation (~3%) and the small ratio of trial participants to the NCDB patients in our analysis.**

**Second, we have extrapolated hospital-level cancer registry data to individual trial participants based on zip code, and we recognize that these pooled, area-based socioeconomic indicators may not reflect the heterogeneity of the communities from which they are sourced, particularly for minority patients who, relative to whites, tend to live in areas that are more socioeconomically diverse while being more racially homogeneous (Erbe BM. Race and Socioeconomic Segregation. *American Sociological Review.* 1975;40(6):801-812).**

**Third, we were unable to assign insurance status to trial participants, and we recognize that some of the patterns of participation observed in our study may be mediated by patient payor status. However, our subgroup analysis of patients aged 65 and over reassured us that findings seen in the full cohort were also observed in the subgroup of older adults for whom Medicare would typically be the primary form of insurance. We also recognize that some states provide Medicaid coverage for the non-trial care of trial participants. But given the expectation that few trial participants would be Medicaid beneficiaries (only 6% of the NCDB control patients had Medicaid) and significant state-by-state variability in Medicaid eligibility, we did not feel it would be appropriate to extrapolate from the data available to us the extent to which state-specific, non-trial Medicaid coverage might have influenced likelihood of trial participation.**

References

1. Hunt KK, Ballman KV, McCall LM, et al. Factors associated with local-regional recurrence after a negative sentinel node dissection: results of the ACOSOG Z0010 trial. *Annals of surgery.* 2012;256(3):428-436.

2. Giuliano AE, McCall L, Beitsch P, et al. Locoregional recurrence after sentinel lymph node dissection with or without axillary dissection in patients with sentinel lymph node metastases: the American College of Surgeons Oncology Group Z0011 randomized trial. *Annals of surgery.* 2010;252(3):426-432; discussion 432-423.

3. Ellis GK, Barlow WE, Gralow JR, et al. Phase III comparison of standard doxorubicin and cyclophosphamide versus weekly doxorubicin and daily oral cyclophosphamide plus granulocyte colony-stimulating factor as neoadjuvant therapy for inflammatory and locally advanced breast cancer: SWOG 0012. *Journal of clinical oncology : official journal of the American Society of Clinical Oncology.* 2011;29(8):1014-1021.

4. Ellis MJ, Suman VJ, Hoog J, et al. Ki67 Proliferation Index as a Tool for Chemotherapy Decisions During and After Neoadjuvant Aromatase Inhibitor Treatment of Breast Cancer: Results From the American College of Surgeons Oncology Group Z1031 Trial (Alliance). *Journal of Clinical Oncology.* 2017;35(10):1061-1069.

5. Buzdar AU, Suman VJ, Meric-Bernstam F, et al. Fluorouracil, epirubicin, and cyclophosphamide (FEC-75) followed by paclitaxel plus trastuzumab versus paclitaxel plus trastuzumab followed by FEC-75 plus trastuzumab as neoadjuvant treatment for patients with HER2-positive breast cancer (Z1041): a randomised, controlled, phase 3 trial. *The Lancet Oncology.* 2013;14(13):1317-1325.

6. Boughey JC, Suman VJ, Mittendorf EA, et al. Sentinel lymph node surgery after neoadjuvant chemotherapy in patients with node-positive breast cancer: the ACOSOG Z1071 (Alliance) clinical trial. *JAMA.* 2013;310(14):1455-1461.

7. Simmons RM, Ballman KV, Cox C, et al. A Phase II Trial Exploring the Success of Cryoablation Therapy in the Treatment of Invasive Breast Carcinoma: Results from ACOSOG (Alliance) Z1072. *Annals of Surgical Oncology.* 2016;23(8):2438-2445.

8. Rosenkranz KM, Ballman K, McCall L, et al. The Feasibility of Breast-Conserving Surgery for Multiple Ipsilateral Breast Cancer: An Initial Report from ACOSOG Z11102 (Alliance) Trial. *Annals of Surgical Oncology.* 2018.

9. Hwang ES, Duong S, Bedrosian I, et al. Abstract GS5-05: Primary endocrine therapy for ER-positive ductal carcinoma in situ (DCIS) CALGB 40903 (Alliance). *Cancer Research.* 2018;78(4 Supplement):GS5-05.

10. Khan SA, DesJardin ES. Optimizing Breast Cancer Management. In: Gradishar WJ, ed. Switzerland: Springer; 2018.

11. Roy V, Pockaj BA, Allred JB, et al. A Phase II trial of docetaxel and carboplatin administered every two weeks as preoperative therapy for stage II or III breast cancer: NCCTG Study N0338. *American journal of clinical oncology.* 2013;36(6):10.1097/COC.1090b1013e318256f318619.

12. Krag DN, Anderson SJ, Julian TB, et al. Sentinel-lymph-node resection compared with conventional axillary-lymph-node dissection in clinically node-negative patients with breast cancer: overall survival findings from the NSABP B-32 randomised phase 3 trial. *The Lancet Oncology.* 2010;11(10):927-933.

13. Margolese RG, Cecchini RS, Julian TB, et al. Anastrozole versus tamoxifen in postmenopausal women with ductal carcinoma in situ undergoing lumpectomy plus radiotherapy (NSABP B-35): a randomised, double-blind, phase 3 clinical trial. *The Lancet.* 2016;387(10021):849-856.

14. SWOG. S9927 Phase III Intergroup. 2003; <http://ncctg.mayo.edu/thebook/Books/Fall_2003/S9927_Full_Report.pdf>. Accessed 14 July 2018.
